# Supplementary material for: Stacking Model‐Based Classifiers for Dealing With Multiple Sets of Noisy Labels
Source: Biom J. 2025 Mar 12;67(2):e70042. doi: 10.1002/bimj.70042 (PMC11898607; doi:10.1002/bimj.70042)
Supplement: Supplementary file 1 — Supporting Information [file BIMJ-67-e70042-s001.zip › density-based-ensemble-model-main/competitors_real_data/netlab-master/netlab-master/help/mlphess.htm]

Netlab Reference Manual mlphess


# mlphess

## Purpose

Evaluate the Hessian matrix for a multi-layer perceptron network.

## Synopsis

```
h = mlphess(net, x, t)
[h, hdata] = mlphess(net, x, t)
h = mlphess(net, x, t, hdata)
```

## Description

`h = mlphess(net, x, t)` takes an MLP network data structure `net`,
a matrix `x` of input values, and a matrix `t` of target
values and returns the full Hessian matrix `h` corresponding to
the second derivatives of the negative log posterior distribution,
evaluated for the current weight and bias values as defined by
`net`.

`[h, hdata] = mlphess(net, x, t)` returns both the Hessian matrix
`h` and the contribution `hdata` arising from the data dependent
term in the Hessian.

`h = mlphess(net, x, t, hdata)` takes a network data structure
`net`, a matrix `x` of input values, and a matrix `t` of
target values, together with the contribution `hdata` arising from
the data dependent term in the Hessian, and returns the full Hessian
matrix `h` corresponding to the second derivatives of the negative
log posterior distribution. This version saves computation time if
`hdata` has already been evaluated for the current weight and bias
values.

## Example

For the standard regression framework with a Gaussian conditional
distribution of target values given input values, and a simple
Gaussian prior over weights, the Hessian takes the form

```
    h = beta*hd + alpha*I
```

where the contribution `hd` is evaluated by calls to `mlphdotv` and
`h` is the full Hessian.

## See Also

`mlp`, `hesschek`, `mlphdotv`, `evidence`

---

**Pages:**
Index


---

Copyright (c) Ian T Nabney (1996-9)
